# Novel Programmed Cell Death as Therapeutic Targets in Age-Related Macular Degeneration?

**DOI:** 10.3390/ijms21197279

**Published:** 2020-10-01

**Authors:** Ming Yang, Kwok-Fai So, Wai Ching Lam, Amy Cheuk Yin Lo

**Affiliations:** 1 Department of Ophthalmology, Li Ka Shing Faculty of Medicine, The University of Hong Kong, Hong Kong, China; hrmeym@hku.hk (M.Y.); hrmaskf@hku.hk (K.-F.S.); 2 State Key Laboratory of Brain and Cognitive Sciences, The University of Hong Kong, Hong Kong, China

**Keywords:** retina, vision, ocular stress, cell damage, homeostasis

## Abstract

Age-related macular degeneration (AMD) is a leading cause of severe visual loss among the elderly. AMD patients are tormented by progressive central blurring/loss of vision and have limited therapeutic options to date. Drusen accumulation causing retinal pigment epithelial (RPE) cell damage is the hallmark of AMD pathogenesis, in which oxidative stress and inflammation are the well-known molecular mechanisms. However, the underlying mechanisms of how RPE responds when exposed to drusen are still poorly understood. Programmed cell death (PCD) plays an important role in cellular responses to stress and the regulation of homeostasis and diseases. Apart from the classical apoptosis, recent studies also discovered novel PCD pathways such as pyroptosis, necroptosis, and ferroptosis, which may contribute to RPE cell death in AMD. This evidence may yield new treatment targets for AMD. In this review, we summarized and analyzed recent advances on the association between novel PCD and AMD, proposing PCD’s role as a therapeutic new target for future AMD treatment.

## 1. Introduction

Age-related macular degeneration (AMD) is a global primary cause of serious blindness [1,2]. In addition to aging, which is the leading factor of AMD [3], the etiology of AMD may also be ascribed to genetics (e.g. CFH and ARMS2 gene) [4], smoking [5,6], nutritional disorders [7], chronic light damage [8], and hypertension [9]. The predominant symptom of AMD patients is progressive central vision loss. 

AMD is classified into non-neovascular and neovascular types. Non-neovascular AMD is further divided into ‘‘early dry’’ and ‘‘late dry’’ AMD. Neovascular AMD, also called ‘‘wet AMD’’, is a late and serious type of AMD. Among the global AMD population, the proportion of non-neovascular AMD is 80–90%, while the percentage of neovascular AMD is 10–15% [10]. Early dry AMD is defined by the presence of medium-size drusen deposition without pigmentary changes and vision loss. ‘‘Late dry’’ AMD, also called geographic atrophy (GA), is a chronic progressive macular degeneration, with sharply demarcated atrophy in the retina, retinal pigment epithelium (RPE), and choriocapillaries. Neovascular AMD is characterized by choroidal neovascularization which starts from choriocapillaris, extending to the Bruch′s membrane. Overall, vision loss is minimal or nonexistent in early-stage AMD, while late-stage AMD patients has vision loss symptoms. 

**Figure 1:**
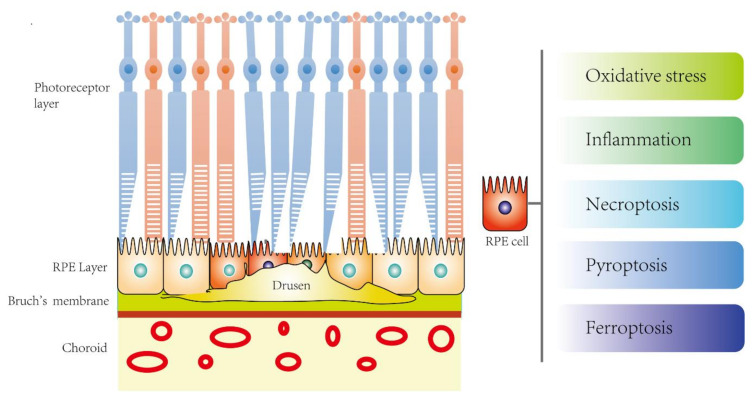
The potential novel disease mechanisms of RPE cell death in age-related macular degeneration (AMD).

Drusen accumulation causing RPE cell damage is the hallmark feature of AMD pathology while oxidative stress and inflammation are the well-known molecular mechanisms. However, the underlying mechanisms behind RPE cell stress in response to drusen deposits are still poorly understood. Programmed cell death (PCD) plays an important role in response to stress and the regulation of homeostasis and diseases. Apart from the classical apoptosis, recent studies also revealed the involvement of novel PCD such as pyroptosis, necroptosis, and ferroptosis, which may contribute to the RPE cell death in AMD (Figure 1).

## 2. Disease Mechanisms of AMD

### 2.1. Pathophysiology

The dominating pathophysiology of AMD is drusen formation, due to RPE’s inability to phagocytose and digest the shredded outer segment of the photoreceptor cells [11]. In the retina, the outer segment of the photoreceptor cells continuously generates residual small bodies as part of their renewal process. Initially, healthy RPE cells [12] have a strong phagocytic ability to remove these residual small bodies [11]. However, due to the decreased phagocytic function in AMD RPE cells, the residual small bodies cannot be cleared timely. As a result, they accumulate in the protoplasm located in the basal part of photoreceptors, eventually deposit in the Bruch’s membrane, and form drusen, leading to macular degeneration [13].

Drusen is a yellowish deposit between the Bruch membrane and RPE cell layer, which can lead to atrophy of RPE cells. Drusen in AMD patients consists of various components. Amyloid beta oligomers in the drusen of AMD patients are toxic to the RPE cells and play a leading role in the pathogenesis of AMD [14].

Interestingly, although drusen accumulates in the retina diffusely, it is the macula that displays the most significant degeneration changes. Firstly, the density of RPE cells in the macula is higher than that in other parts of the retina [15]. When the RPE loss happens at the macula, it needs to be replenished by the adjacent RPE cells [16,17]. Secondly, the expression of ARMS2, a risk gene of AMD, is higher in macular RPE cells, making them more likely to develop degenerative changes [18]. Overall, due to the differences in the regional location and genetic background between macular and peripheral RPE cells, their capabilities in anti-oxidation, immune response modulation, and tissue repair will vary [19,20,21]. As more drusen accumulates, this can induce atrophy of RPE cells, and as a result, photoreceptor cells lose the support from RPE cells, leading to macular degeneration [13]. These processes together contribute to the formation of the dry type of AMD. 

A recent meta-analysis showed that a high level of plasma interleukin-6 (IL-6) is associated with neovascular AMD and geographic atrophy, suggesting that the late stages of AMD are accompanied by chronic low-grade inflammation and the therapeutic potential of targeting systemic IL-6 [22]. In AMD, drusen accumulation can also trigger inflammation, where inflammatory cells are then drawn to the retina [23]. Microglia, macrophages, or other cells expressing IL-6 were significantly increased in the retina of donors with geographic atrophy, suggesting the activated inflammatory activities [24]. These inflammatory cells and RPE cells secrete growth factors that promote the growth of blood vessels under the RPE layer. One of the major growth factors released is vascular endothelium growth factor (VEGF) [25], which can diffuse into the choroid, contributing to the growth of new blood vessels [12]. Once the Bruch’s membrane breaks, the abnormal newly-formed choroidal neovascularization (CNV) could penetrate the ruptured Bruch’s membrane, extend to the sub-RPE layer, and proliferate under the neurosensory retina [26]. Due to the fenestration of the abnormal neovascular blood vessel wall, fluid leakage and hemorrhage occur, which may lead to a series of secondary pathological changes, including serous macular detachment [27]. These pathological processes convert non-neovascular AMD [28] into neovascular AMD (wet AMD), causing rapid central vision loss.

In general, oxidative stress, inflammation, and loss of RPE cells and photoreceptors lead to persistent neurodegenerative cellular death [29]. However, the mechanism of late dry AMD, also known as GA, is still not fully understood. 

### 2.2. Molecular Mechanisms

Oxidative stress and inflammation are considered to be involved in the molecular mechanisms of AMD pathogenesis, causing progressive RPE damage [29,30]. Reactive oxygen species (ROS) including superoxide anion, hydroxyl radical, and hydrogen peroxide play a dominant role in oxidative damage. ROS are generated through the mitochondrial electron transport chain in normal metabolism. However, the produced ROS will be quickly cleared by its own anti-oxidative stress metabolism through superoxidase dismutase and glutathione, thereby maintaining homeostasis. 

Aging is the primary factor of AMD, and as the age increases, the production of ROS and subsequently the level of oxidative stress in the RPE cells also increase. Moreover, the activities of antioxidant enzymes such as superoxide dismutase (SOD), glutathione s transferase (GST), and glutathione (GSH) that play important roles in oxidative stress neutralization decrease with the aging process [31]. 

In the retina, RPE and photoreceptor cells require a large supply of oxygen and nutrients for their metabolism and function, thus generating excess ROS [32]. Being constantly produced, the ROS can damage intracellular organelles such as mitochondria and lysosomes. Mitochondrial damage can further induce and generate additional ROS, resulting in a vicious cycle that causes further RPE damage [31]. As RPE cells are terminally differentiated cells, they cannot regenerate once injured. The continual presence of ROS is detrimental to RPE’s survival. 

Oxidative stress also aggravates drusen production and stimulates the generation of histocompatibility complex, C-reactive protein, and other inflammatory factors and complements [33]. In addition, the components of drusen are potent stimulants of chronic inflammation, forming a pro-inflammatory microenvironment in the eye, stimulating the expression of inflammatory mediators from the RPE or choroid vascular smooth muscle cells, thereby promoting the occurrence of inflammation and the development of AMD [34]. An example is Amyloid beta_1-40_ oligomers, one of the major components of drusen [14]. These mechanisms cause RPE cell damage and therefore insufficient ability to clean up the drusen in the retina. At this time, macrophages are activated to phagocytose the drusen complex to assist with RPE tissue repair [35]. However, the dead macrophages after phagocytosis and other substances can result in increased VEGF level, which eventually causes neovascularization, thereby developing into wet AMD [36,37,38,39].

Wet AMD signaling pathways are concentrated on angiogenesis. VEGF, the first molecule that was found to mediate angiogenesis, was discovered in 2000 [40]. VEGF-A, a family member of VEGF, plays a major role in angiogenesis, predisposing the development into the wet AMD. It initiates three main signaling pathways, including MAPK-p38 [41], PI3K-AKT [42], protein kinase B, and PLCγ [43] by binding with the VEGF receptor. Later studies have also found that the metalloproteinases (MMP) families [44,45] and thrombospondin-1 (TSP-1) [46,47] play different roles in disrupting the homeostasis between angiogenic and anti-angiogenic factors, contributing to pathological neovascularization. In addition to the above molecules, a recent review using Search Tool for the Retrieval of Interacting Genes (STRING) analysis identified and summarized potential angiogenesis-related proteins. These proteins include the platelet-derived growth factor family, pigment epithelium-derived factor, hepatocyte growth factor, epidermal growth factor, angiopoietins, endothelins, fibroblast growth factor family, transforming growth factor-beta, the angiopoietin-like family, the galectin family, hypoxia-inducible factors, insulin-like growth factors, cytokines, and integrins, providing new targets to tackle wet AMD [48].

### 2.3. RPE In Vitro AMD Studies

RPE is a layer of pigment cells located adjacent to and nourishing the photoreceptors in the retina [49]. It is closely connected with the choroid beneath it and the retinal photoreceptors above it. As the primary pathogenesis of AMD is located at RPE cells, they are considered as the most suitable research tool for the AMD in vitro model [50]. 

To date, in vitro studies mainly focus on dry AMD. The ARPE-19 cell line and primary human RPE cells are widely used. Several agents have been used to induce RPE cell death as an in vitro model of AMD. Based on its presence in drusen, amyloid beta_1-40_ oligomers are considered the most suitable inducer to mimic AMD in mechanistic studies. Most importantly, post-mortem examination in AMD patients proved that amyloid-beta_1-40_ is a major component of drusen [39]. Using amyloid beta_1-42_ oligomers to induce AMD, a study found that autophagy played a protective role [30]. However, amyloid beta_1-42_ was mostly involved in the pathogenesis of Alzheimer’s disease while amyloid beta_1-40_ is considered AMD specific. This limits the validity of the AMD in vitro model. Two other studies also used amyloid beta_1-40_ oligomers to build the AMD model, but in lipopolysaccharide (LPS)-primed ARPE-19 cells [51,52]. As LPS is not associated with AMD, this in vitro model is possibly invalid to mimic AMD. Instead, Amyloid beta_1-40_ oligomers-exposed non-LPS-primed RPE cell [53] may more likely mimic AMD. Another study showed that amyloid beta_1-40_ oligomers at a high concentration (25 μM) lowered ARPE-19 cell viability after 24 hours of stimulation [38]. These studies suggested that the use of amyloid beta_1-40_ oligomers is suitable to provide an in vitro model for studying RPE cell death and AMD pathogenesis. 

Besides using amyloid beta_1-40_ oligomers, oxidative stress upon RPE cells may also resemble AMD conditions in vitro. Studies showed that a low dose of sodium iodate significantly decreased phagocytotic activity, cellular acidity, and autophagy, leading to RPE cell degeneration [54,55], while a high dosage of sodium iodate increased the expression of pentraxin 3, thereby accelerating RPE cell death [56]. Another in vitro model was the application of *tert*-butyl hydroperoxide on human fetal RPE and APRE-19 cells [57]. Moreover, a hydrogen peroxide-induced AMD cellular model has also been used to test the therapeutic efficacy of piceatannol [58], scutellarin [59], and berberine [60].

## 3. Prevention and Intervention Strategies of AMD

### 3.1. Dry AMD

Early dry AMD may not be immediately sight-threatening; it is usually asymptomatic but later will develop symptoms, including visual distortion and reduced central vision. At present, no standard treatment for dry AMD is available in the world. However, several non-drug and drug intervention strategies have been recommended. Initially, maintaining a healthy lifestyle, including a balanced diet, regular exercise, wearing sunglasses, and quitting smoking, reduce the risk of dry AMD progression. In 2001, a randomized, placebo-controlled, age-related eye disease study (AREDS) showed that high-dose supplementation with vitamins C and E, beta carotene, and zinc significantly reduced the rate of visual acuity loss [61]. Studies also suggested that antioxidants including vitamins, lutein, and zeaxanthin may reduce the transformation of dry AMD to the reversible wet AMD [62]. Moreover, long-term supplementing of vitamins C and E, β-carotene, and zinc could significantly reduce the risk of the development of advanced AMD [63]. In 2014, AREDS with a ten-year follow-up reported that both age and the degree of drusen accumulation increased the risk of progression to advanced AMD [64]. AREDS2 further reported that the effect of lutein or zeaxanthin supplementation on AMD was better than beta-carotene [65]. The antioxidants reduce RPE damage by limiting the generation of free radicals, protecting photoreceptors and acting as retinal tissue nutrients [66]. In addition, recent single-center studies showed that photobiomodulation, a recently proposed light therapy, improved symptoms and reversed pathological changes (drusen formation) without causing harmful effects [67,68], suggesting a novel strategy for the earlier stage of AMD.

### 3.2. Wet AMD

The current treatments for wet AMD are anti-VEGF drugs, while photodynamic therapy is considered a second-line therapy today. According to the American Academy of Ophthalmology, anti-VEGF treatment improves vision in about one third (1 out of 3) of patients who receive it. For a vast majority (9 out of 10), anti-VEGF can at least stabilize vision [69]. However, there are still limitations in the current anti-VEGF treatment; the vision of around two-third of the patients receiving anti-VEGF treatment could not be improved. Therefore, a long-term visual benefit has not been achieved yet, and most treatments are mainly focused on delaying the progression of the diseases.

#### 3.2.1. Anti-VEGF Drugs 

Anti-VEGF drugs are typically delivered through intravitreal injection. Recently, a report by the American Academy of Ophthalmology reviewed 28 clinical trials of Anti-VEGF drugs, suggesting considerable safety and efficacy over the two years’ treatment for wet AMD [25]. Anti-VEGF drugs, such as ranibizumab and bevacizumab, bind to all VEGF isoforms to inhibit angiogenesis, thereby limiting the development of CNV [70]. Nevertheless, the effectiveness can only be sustained by continual periodical intraocular injection. In spite of the efficacy, some cases still showed poor vision outcomes after long-term anti-VEGF therapy. Moreover, several clinical trials also reported that GA developed in neovascular AMD patients after continual treatment with ranibizumab or bevacizumab [70,71,72,73]. One study even showed that the cumulative incidence of GA increased from the first (12%) to the fifth year (38%) of treatment. However, the percentages of GA still were significantly lower than the improved rates of visual acuity in these studies [74,75,76,77], suggesting anti-VEGF drug is beneficial as a long-term therapy for neovascular AMD. Yet, the development of alternative therapies is warranted in the future. 

#### 3.2.2. Photodynamic Therapy (PDT) 

PDT combines a light-activated drug with a low-energy laser. The photosensitive drug (verteporfin) is injected through intravenous infusion and travels to the abnormal vessels under the central macula [78]. It then attaches to molecules such as low-density lipoprotein, integrin, and monoclonal antibodies that are commonly found in rapidly growing cells, for example new endothelial cells in neovascular AMD [79]. Finally, a low-energy laser light is focused directly onto the abnormal vessels, which activates the drug, causing damage specifically to the abnormal blood vessels. PDT can effectively delay the progress of AMD and reduce patients from severe vision loss. Although several treatments are usually required, PDT has largely replaced thermal laser therapy, which may cause permanent damage to the normal retina. However, the recurrence of the neovascularization is high in both PDT and thermal laser therapy. Moreover, PDT cannot restore the damaged macula, and has the risk of causing vascular occlusion, and thereby can further impair the vision [80].

#### 3.2.3. Stem Cell Therapy 

A stem cell is a type of unlimited self-renewal cell that can differentiate into other types of cells. Induced pluripotent stem cells (iPSCs) and human embryonic stem cells (hESCs) can be differentiated into retinal cells, sharing the same characters with the original ones at both genetic and functional levels. Using stem cells, the damaged retina can be repaired and substituted by the paracrine effect [81]. Another advantage of using stem cells is that they are immune friendly to the host. The risk of immunological rejection is significantly lower compared with RPE transplantation. 

On-going clinical trials on stem cells for AMD treatment have been conducted in different countries [82,83,84,85,86]. hESCs and iPSCs derived from AMD patients were first used in the AMD clinical trials in the United States and Japan, respectively [84,87]. The preliminary and Phase I/II clinical studies showed that hESCs-derived RPE cell therapy has safely and effectively promoted general and peripheral vision, visual acuity and near/distance activities in AMD patients [84,85]. Moreover, in 2018, a study developed a bioengineered retinal pigment epithelial monolayer to deliver hESCs-derived RPE [83] and conducted a Phase I clinical study in advanced stage AMD patients [82]. These new technologies contribute to novel therapeutic strategies for AMD and help to improve visual acuity [82,83]. However, the sample sizes of both studies are relatively small, while the observation period in one study was only 12 months [82], which is not sufficient for safety and tumorigenicity evaluation. In fact, although stem cell therapy is a regenerative treatment option, it is costly in time and money. 

Taken together, to date, some but not all pathogenic processes of AMD have been revealed and therapies for preventing these processes are being used. In spite of the implementation of current therapies, the reoccurrence rate is high and no existing therapeutic strategy can cure the disease, which may lead to excessive health care expenditures and substantial socio-economic burden worldwide. Consequently, improved understanding of the underlying mechanisms in RPE response after drusen exposure that allows exploration of novel strategies to prevent and tackle AMD is urgently needed.

## 4. Overview of Novel Programmed Cell Death (PCD)

**Figure 2:**
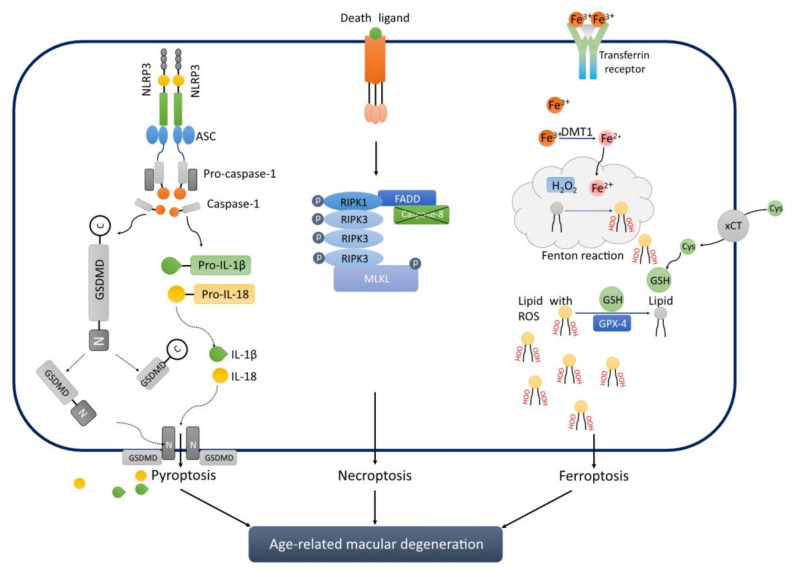
A schematic diagram of the association between novel programmed cell death and AMD.

Programmed cell death (PCD) plays an important role in response to stress and the regulation of homeostasis and diseases. Apart from the classical apoptosis, recent basic science studies also discovered novel PCD such as pyroptosis [53], necroptosis [88] and ferroptosis [57], which may contribute to the RPE cell death in AMD (Figure 2). A comparison of the three novel programmed cell deaths has been listed in Table 1. These novel PCD pathways may yield new treatment targets for the AMD [89].

### 4.1. Pyroptosis

Pyroptosis is a newly discovered inflammasome-related cell death pathway 2001 [94]. Cell swelling, membrane blebbing, rupture, and pore formation with pyroptotic bodies are the key morphological changes when pyroptosis happens [95,96]. Based on the signaling differences, pyroptosis is classified into classical and non-classical types.

#### 4.1.1. Classical Type of Pyroptosis

When stimulated by bacteria and viruses, the pattern recognition receptors in the infected cells act as sensors to recognize these signals and in turn combine with the precursor of caspase-1 through the adaptor protein ASC to form a polyprotein complex, which activates caspase-1 [97,98]. On one hand, caspase-1 cleaves Gasdermin D to form a peptide containing the nitrogen-terminal active domain of Gasdermin D (GSDMD-N), which induces cell membrane perforation, cell rupture, the release of contents, and inflammatory response [90,96]. On the other hand, activated caspase-1 cleaves the precursor of interleukin (IL)-18 to generate active IL-1β and IL-18, which are released out of the cell. Inflammatory cells are in turn recruited and aggregate, expanding the inflammatory response [99].

#### 4.1.2. Non-Classical TYPE of Pyroptosis

Caspase-4, 5, and 11 launch the commencement of non-classical type of pyroptosis pathway when cells are exposed to stress. The activated caspase-4, -5, and -11 cleave Gasdermin D to form a peptide containing the GSDMD-N [96]. On the one hand, it induces cell membrane perforation, cell rupture, the release of contents, and inflammatory response [99]. On the other hand, the activated GSDMD-N also promotes the activation of caspase-1, which cleaves the precursors of IL-1β and IL-18 to form active IL-1β and IL-18, and releases them to the outside of the cell, recruiting inflammatory cells to gather and expand the inflammatory responses [100]. These later processes are similar to those in classical pyroptosis, but the initiators are caspase-4, 5, and 11 instead of caspase-1.

### 4.2. Necroptosis

Necroptosis is also a novel programmed cell necrosis recently discovered in 2005 [101]. This is the first novel programmed cell death that does not depend on the activation of caspases rather based on the formation of necrotizing bodies. RIPK1, RIPK3, and MLKL are the main molecules involved in necroptosis [92]. Necroptosis shares some of the morphological features with pyroptosis, such as cell swelling, pore formation, and rupture, but displays no membrane blebbing.

Apoptosis and necroptosis share the same receptors, such as tumor necrosis factor (TNF) receptor, which can lead to extrinsic apoptosis by recruiting caspase-8 [102]. However, many studies proved that inhibition of caspase-8 shifted apoptosis to necroptosis because of the activation of RIPK3 and MLKL [103,104,105,106]. Therefore, necroptosis is a different mode of programmed cell death, and it is a caspase-8 independent apoptosis pathway in which caspase-8 was blocked [107]. 

Ser227 in RIPK3 is phosphorylated during necroptosis. Ser227 phosphorylation of RIPK3 is necessary for the activation of MLKL, which acts as an effector protein downstream of RIPK1 and RIPK3. These molecules are part of complex IIb, also called necrosomes. The phosphorylation of MLKL allows MLKL oligomerization and translocation to the plasma membrane, which interacts with phosphatidylinositol to induce membrane permeation. MLKL-induced plasma membrane permeation directly forms pores and leads to Ca^2+^ or Na^+^ influx, releasing cell-damage-related molecular patterns (cDAMP), such as mitochondrial DNA (mtDNA), high-mobility group protein B1 (HMGB1), interleukin (IL) -33, IL-1α, and ATP.

### 4.3. Ferroptosis

Ferroptosis is the latest programmed cell death pathway discovered in 2012 [108]. It is an iron-dependent non-apoptotic programmed necrosis playing important roles in the pathogenesis of multiple diseases [109]. It starts with iron accumulation and overload, followed by the Fenton reaction to generate lipid ROS, thereby causing damage to the cell membrane [109]. The up-regulation of COX-2, ACSL-4, PTGS2, and NOX1 and down-regulation of GPX-4 and FTH1 contribute to ferroptosis. 

The process of ferroptosis is accompanied by the accumulation of a large amount of iron ions and lipid peroxidation at the same time. When compared with the normal cells, there is a decreased mitochondrial size where the mitochondrial membrane shrinks, accompanied by a decreased mitochondrial crest and increased double-layer membrane density. Ferroptosis occurs due to the imbalance between the production and degradation of lipid ROS in cells. Ferroptosis inducers directly or indirectly act on glutathione peroxidase (GPXs) through different pathways (including the regulation pathway of iron homeostasis, the RAS pathway and the cystine transport pathway), leading to ROS accumulation, failure of cellular antioxidant capacity, and cell oxidative damage. A plethora of substances and external conditions can trigger ferroptosis. For example, the small molecule erastin inhibits the cystine-glutamate exchanger on the plasma membrane and reduces the cell’s acquisition of cystine, thereby blocking GPX4’s substrate-glutathione peptide synthesis, which in turn triggers the accumulation of membrane lipid ROS and ferroptosis [110].

Overall, ferroptotic cell death is a disorder of lipid oxide metabolism in the cell. The lipid oxide metabolism is abnormal under the catalysis of iron ions. The lipid active oxygen accumulates when the antioxidant capacity of the cell is weakened, causing an imbalance of intracellular redox and inducing cell death.

## 5. Novel PCD is Associated with AMD 

### 5.1. Pyroptosis and AMD

NLRP3 is the initial step of pyroptosis activation, followed by ASC and caspase-1, which cleaves Pro-IL-1β and IL-18, and activates GSDMD thereby triggering inflammation and cell death. In 2013, NLRP3 was first detected in RPE and drusen in AMD patients. Using human RPE cells, the study also provided the evidence that lysosomal destabilization triggered NLRP3-mediated inflammation [111]. However, markers of pyroptosis were not examined in this study. Using caspase-1 knock out mice, a study showed increased photoreceptor survival and better-preserved retinal function with reduced inflammatory activity in mouse eyes, indicating the potential of targeting caspase-1 in AMD treatment [112]. It was later shown that not only NLRP3 inflammasome was activated, but the expressions of GSDMD-N, IL-1β and IL-18 were also significantly increased in an amyloid β_1-40_ oligomers-induced AMD in vivo model, suggesting the activation of pyroptosis [113]. These in vitro and in vivo studies confirmed that pyroptosis plays an important role in the pathogenesis of AMD, while inhibiting this target may benefit the prognosis of AMD. 

### 5.2. Necroptosis and AMD

Oxidative stress is an important molecular mechanism of AMD (and a primary cause of necroptosis) leading to RPE damage [114]. In 2016, Hanus et al. [88] for the first time showed the existence of necroptosis in a sodium iodate induced-AMD in vitro model. They also showed that only the inhibitors targeting RIPK1 and RIPK3 kinases displayed the most prominent efficacy of increasing ARPE-19 survival when exposed to sodium iodate. Yet, this is the only study that described the presence of necroptosis in the AMD in vitro model. Further studies are needed to confirm the relationship of oxidative stress and necroptosis in the pathogenesis of AMD.

### 5.3. Ferroptosis and AMD

In 2019, Totsuka and his colleagues used *tert*-butyl hydroperoxide-induced fetal RPE and ARPE-19 cell damage as two AMD in vitro models. In their study, they found that RPE cells were damaged through lipid peroxidation, GSH depletion, and iron accumulation, suggesting the occurrence of ferroptotic cell death in these in vitro models [57]. GSH plays an important role in eliminating ROS and ferroptosis. Indeed, a recent study found that the depletion of GSH induced ferroptosis in APRE-19 cells [115]. Although GSH deficiency is not considered as a model of AMD, the study still demonstrated the presence of ferroptosis in RPE cells. 

### 5.4. Aging and Novel PCD in AMD?

AMD is a disease in the aged population. The prevalence of RPE depigmentation, pigmentary abnormalities, and drusen severity increase with the aging process, thereby aggravating the formation and development of AMD [116]. A ten-year follow-up clinical study also showed that aging promotes the transfer from early- to advanced-stage of AMD [64]. Indeed, aging increased the prevalence of choroidal neovascularization and geographic atrophy [116]. 

Aging is a complex biological process with progressive loss of tissue and organ function. In the oxidative stress theory of aging, reactive oxygen and nitrogen species [117] [117] result in oxidative damage to macromolecules (lipids, DNA, and proteins), which accumulates over time and eventually leads to age-associated functional loss [118]. Increased RONS may also lead to cellular senescence, an irreversible physiological process that stops cell division to prevent proliferation-associated damages to the cell. In addition, aging causes upregulation of nuclear factor (NF)-κB signaling, cytokines/chemokines, endoplasmic reticulum (ER) stress, inflammasome, and lipid accumulation, eventually leading to chronic inflammation [119]. The activation NF-κB pathway and age-related inflammation may also be contributed by disruptions in PCD including apoptosis during aging [120]. Although currently there is no direct evidence linking aging and novel PCD, the disease microenvironment factors such as oxidative stress, inflammatory activity, and drusen accumulation generated by aging in AMD could lead to novel PCD.

## 6. Novel PCD: New Future Therapeutic Targets for AMD?

Based on the evidence summarized above, it is clear that novel PCD contributes to RPE death and pathogenesis of AMD. Therefore, the possibility of it becoming a candidate for a therapeutic target of AMD is worth more investigations. 

Pyroptotic activity was firstly discovered in a Long Evans rats AMD model [113]. In 2020, a subsequent study also found pyroptosis involved in amyloid β_1-40_ oligomers-induced human RPE cells, an in vitro AMD model [53]. More importantly, the observed increased expression of NLRP3 inflammasome, membrane GSDMD-N, as well as secretion of IL-1β and IL-18 could be significantly suppressed by *Lycium barbarum* polysaccharides (LBP) treatment, accompanied by a noticeable morphological recovery, suggesting a protective effect of LBP on an AMD in vitro model by suppressing pyroptosis [53]. Another study [121] demonstrated that Baicalin, an active component of the radix of *Scutellaria baicalensis* [122], alleviated amyloid β_1-42_ oligomers-induced ARPE-19 cell pyroptosis. Moreover, overexpression of tripartite motif-containing protein 31 (TRIM31) could suppress ox-LDL induced-pyroptosis in ARPE-19 cells [123]. TRIM31 is well-known to regulate innate immune responses negatively [123]. These findings suggested pyroptosis as an effective therapeutic target for AMD.

As oxidative stress triggers necroptosis, thereby aggravating the development of AMD [114], targeting necroptosis might be an alternative in addition to suppressing oxidative stress, as cell death is harder to reverse via oxidative stress once it happens. Because of the limited therapeutic options nowadays, future studies should evaluate necroptosis as a potential novel target for AMD. Indeed, owing to its caspase-independent property, necroptosis contributes to many neurodegenerative diseases. Necroptosis inhibitors exemplified the treatment effect in neurodegenerative diseases [124], including amyotrophic lateral sclerosis [125]. 

Although currently there is no study showing the effect of any drug/agent on ferroptosis in an AMD model, suppressing ferroptosis has shown effectiveness in other neurodegenerative diseases recently. In an aging model of presbycusis [126], increased expression of transferrin receptor 1, iron accumulation, and malondialdehyde and a decreasing level of glutathione and superoxide dismutase were observed, suggesting an activation of ferroptotic activity. Moreover, both iron chelator deferoxamine and knockdown of iron regulatory protein 2 significantly repressed ferroptosis, suggesting ferroptosis as a promising therapeutic target for neurodegenerative disease. Therefore, it is worth investigating the treatment efficacy on AMD by targeting ferroptosis.

Taken together, with the increasing findings of association of novel PCD such as necroptosis, ferroptosis and pyroptosis with AMD pathogenesis, there is an urgent and promising need to evaluate the efficacy of the new treatment targets thereby filling the gap in the current limited therapeutic options of AMD. 

## 7. Conclusion and Future Remarks

Based on the evidence from RPE cell damage in AMD in vitro and preclinical models as well as AMD clinical studies, we hypothesize that novel PCD is likely to serve as a new therapeutic target for AMD. Future drugs could be designed for inhibiting or blocking these novel PCD pathways in order to achieve effective intervention and improved prognosis. To date, the more promising pathway would be pyroptosis. However, some evidence has shown that oxidative stress triggers both necroptosis and ferroptosis, thereby aggravating RPE damage in AMD in vitro models. The association of these two pathways with RPE death and AMD pathogenesis remains to be confirmed in future studies. 

## Figures and Tables

**Table 1:** Comparison among Novel Programmed Cell Death Pathways.

Type	Morphology	Activated/Increased Molecules	Inactivated/Decreased Molecules	Type of Cell Membrane Pores	References
Pyroptosis	Cell swelling Membrane blebbing Membrane pore Membrane rupture Pyroptosis bodies	NLRP3, ASC, Pro-caspase-1, and Gasdermin D	N/A	Gasdermin D-N-dependent	[90,91]
Necroptosis	Cell swelling Membrane pore Membrane rupture Necrotizing bodies Nucleus chromatin condensation	RIPK1, RIPK3 and MLKL	Caspase-8	MLKL-dependent	[92]
Ferroptosis	Membrane vacuolated Membrane rupture Membrane density increase Cytoplasm rounding-up	Iron accumulation Lipid reactive oxygen species	GPx-4, GSH, xCT	Lipid reactive oxygen species-dependent	[93]

Pyroptosis, necroptosis, and ferroptosis are novel programmed cell death pathways, which are likely new mechanisms and therapeutic targets for AMD. NLRP3: nod-like receptor protein 3. ASC: apoptosis-associated speck-like protein containing a caspase recruitment domain; GSDMD: Gasdermin D; RIPK: receptor-interacting interacting protein kinase; MLKL: mixed lineage kinase domain-like protein; FADD: Fas associated via death domain; DMT1: divalent metal transporter 1; GSH: Glutathione; Cys: Cystine; xCT: cystine/glutamate antiporter; GPx-4: Glutathione peroxidase 4.
